# GIS and Remote Sensing Use in the Exploration of Lyme Disease Epidemiology

**DOI:** 10.3390/ijerph121214971

**Published:** 2015-12-01

**Authors:** Esra Ozdenerol

**Affiliations:** Department of Earth Sciences, University of Memphis, Memphis, TN 38152, USA; eozdenrl@memphis.edu

**Keywords:** Lyme disease, tick habitat, geographic distribution, risk modeling, spatiotemporal pattern

## Abstract

Given the relatively recent recognition of Lyme disease (LD) by CDC in 1990 as a nationally notifiable infectious condition, the rise of reported human cases every year argues for a better understanding of its geographic scope. The aim of this inquiry was to explore research conducted on spatiotemporal patterns of Lyme disease in order to identify strategies for implementing vector and reservoir-targeted interventions. The focus of this review is on the use of GIS-based methods to study populations of the reservoir hosts, vectors and humans in addition to the spatiotemporal interactions between these populations. New GIS-based studies are monitoring occurrence at the macro-level, and helping pinpoint areas of occurrence at the micro-level, where spread within populations of reservoir hosts, clusters of infected ticks and tick to human transmission may be better understood.

## 1. Introduction

Lyme disease (LD) is the most common tick-borne disease in the temperate zones of the Northern Hemisphere [1]. The process of its extension beyond its endemic foci is predicted to accelerate with climate change. Research modeling climate-change scenarios anticipate climate change will result in geographic distribution of vectors expanding northward as the earth warms, and they specifically forecast the retraction of vectors from the southern U.S. into the central U.S. and the emergence and reemergence of LD in various regions of Canada [2]. Climatic variables and climate change (*i.e.*, warming climate) are likely associated with tick survival and their geographic occurrence [3]. Dispersion of infected ticks by migratory birds could infect wildlife populations in the new frontiers and could introduce endemic cycles of infection into newly established reproducing populations of the tick vector [4]. Human behavior is likely to be affected by climate change which will alter the interaction with vectors and the transmission of diseases they carry to humans. The relationship of density estimates of vectors to human incidence of LD are strongest in high-prevalence areas and varies by region due to the distribution of pathogens and their reservoir hosts’ habitat and climate preferences [5].

The pathogen *Borrelia* known to cause Lyme disease has at least 37 known species, 12 of which are Lyme related, and an unknown number of genomic strains [6]. The strains differ in clinical symptoms as well as geographic distribution [7]. The common causative LD agent in North America, *Borrelia burgdorferi* is transmitted from mammal to mammal (small sized, ground dwelling vertebrate hosts) by ticks of genus *Ixodes scapularis and Ixodes pacificus*. In Europe and Asia, *Borrelia afzelii, Borrelia garinii and Borrelia valaisiana* are the most abundant species [8]. The primary vector species is *Ixodes ricinus* in Europe [9], and *Ixodes persulcatus* in Asia [10]. The pathogen cycles between wild animal hosts and vectors. Humans are accidental dead-end hosts. Typical symptoms include fever, headache, fatigue, and a characteristic skin rash called erythema migrans (EM). This rash occurs in approximately 70%–80% of infected persons and begins at the site of a tick bite. Some patients develop additional EM lesions in other areas of the body several days later [11]. If left untreated, infection can spread to joints, the heart, and the nervous system [12]. Although symptoms have been observed since the 19th century, systematic surveillance for Lyme disease was first initiated in 1982 by the U.S. Centers for Disease Control and Prevention [13]. The impact on personal health is noteworthy and the direct medical cost associated with LD in the United States is estimated at 2.5 billion dollars annually [14]. The increased incidence of reported Lyme disease likely is due to improved awareness and recognition of the disease, as well as to an actual increase in incidence and geographic spread. Currently, there is no vaccine available for human use but several are available for veterinary use [15].

LD could involve the same agent *Borrelia burgdorferi* but different vectors and hosts in different regions in the United States. In the northeastern United States, the white–footed mouse is the primary wildlife reservoir host responsible for infecting ticks (*Ixodes scapularis*), also known as the deer tick [16]. Some infections that occur in the northern California and the upper Pacific Northwest are transmitted by *Ixodes pacificus,* the western black-legged tick, and the main reservoir host is the dusky footed wood rat. The main vector species in European countries are *Ixodes ricinus.* The main host of *Ixodes ricinus* is the roe deer; although not a reservoir, it plays an important role of maintenance and co-feeding for ticks.

In the United States, the proportion of ticks infected with *Borrelia burgdorferi* varies greatly both by geographic area and by the stage of the tick in its three-stage life cycle (*larva, nymph and adult*). Newly hatched larval ticks take a blood meal during the summer of the first year, during which they can acquire infection, and after molting into a nymph (immature ticks less than 2 mm), they take a second blood meal the following year, during which they can transmit infection [17]. Most humans are infected through the bites of nymphs. Humans acquire the disease mainly during the months of May and July after contact with the vector during outdoor activities in tick habitat, which is characterized by close-canopy deciduous and mixed forest [5]. In endemic areas, persons who have either occupational or recreational exposure to tick-infested woodlands or fields are at increased risk of LD, while there is also substantial risk on the lawns of suburban homes that border wooded areas [18]. Adult ticks can also transmit LD bacteria, but they are much larger and may be more likely to be discovered and removed before they have had time to transmit the bacteria. Adult *Ixodes* ticks are most active during the cooler months of the year [19].

In the United States most cases of LD occur in coastal and riparian regions of southern New England, southeastern New York, New Jersey, eastern Pennsylvania, eastern Maryland, Delaware, and parts of Minnesota and Wisconsin [5]. The incidence in the ten states with the highest numbers of cases averaged 302 cases per 100,000 persons between years of 1991 and 2006 [20]. In Canada, Lyme disease became nationally notifiable in 2009. The number of cases reported in Canada has more than doubled in four years, from 144 in 2009 to 338 in 2012, which is an increase in incidence from 0.4 to 1.0 cases per 100,000 population. Endemic LD occurs in British Columbia and in the eastern and central Canadian provinces of Nova Scotia, New Brunswick, Quebec, Ontario and Manitoba where it is continuing to emerge [21].

**Figure 1:**
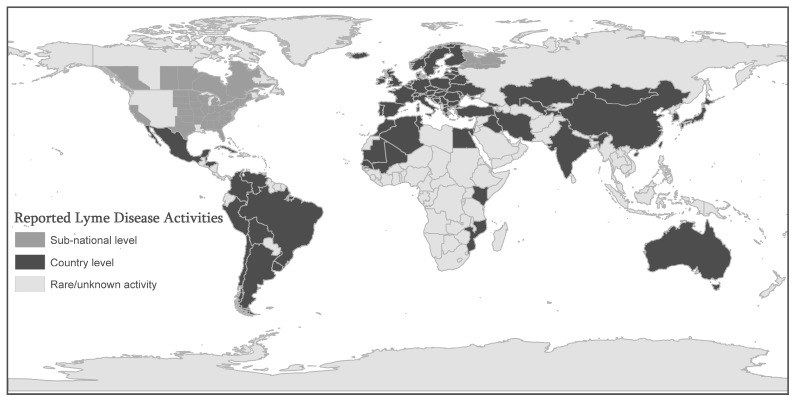
Geographic Extension of Lyme Disease (LD) activities. Figure 1 shows that Lyme Disease (LD) has extended to many countries around the world beyond the endemic foci. Reported LD activities that were mapped include diagnosed cases as well as infected ticks, infected animals, and seropositive human samples. The dark gray shading signifies countries with (at least) some reported LD activity, and the presence of activity is known only at the country level. The lighter gray shading represents areas in which Lyme disease has been reported at the sub-national level in particular regions of some countries. The lightest gray represents counties with rare or unknown activity. This map is compiled from various resources such as published articles reviewed in this paper, Lyme Disease Association, Inc. [30], and World Health Organization websites [31].

In Europe, most cases occur in the Scandinavian countries and in central and eastern Europe, especially in Germany, Belgium, Austria, Slovenia, and Czech republic between the years of 2009 and 2012 [22]. The highest annual incidence is 80 cases per 100,000 persons or higher reported for Slovenia (155/100,000), Austria, Southern Sweden, Netherlands and Switzerland. Incidence lower than 20 cases per 100,000 persons have been reported in France and Poland [23]. The lowest incidences are in UK (0.7/100,000) and Ireland (0.6/100,000). Cases have been reported in over 60 countries and endemic foci in North America, Europe, and Asia [24]. Figure 1 shows that LD has extended to many countries around the world beyond the endemic foci. Reported LD activities that were mapped include diagnosed cases as well as infected ticks, infected animals, and seropositive human samples. The dark gray shading signifies countries with (at least) some reported LD activity, and the presence of activity is known only at the country level. The lighter gray shading represents areas in which Lyme disease has been reported at the sub-national level in particular regions of some countries. The lightest gray represents counties with rare or unknown activity.

In 1990, CDC recognized LD as a nationally notifiable disease and developed a national case definition [25]. A case is confirmed if the person has the skin lesion erythema migrans or if at least one late manifestation of disease is present and the case is laboratory-confirmed. Laboratory confirmation is recommended for persons with no known exposure. Exposure is defined as having been in a county where LD is endemic within 30 days before the onset of erythema migrans [26]. The geographic standard by which the disease is endemic to a county is determined by at least two confirmed cases that have been previously acquired, or established populations of a known tick vector that are infected with *Borrelia burgdorferi.* This geographic standard could be problematic for some regions where erythema migrans and other LD symptoms have been observed in patients who live in areas where there is lack of definitive evidence of transmission to humans, especially in the southeastern United States [27].

Case definitions change over time and updated to assist clinicians in the accurate diagnosis of LD. Variation in case definition by country could make it difficult to compare the number of cases in different countries and regions of the world. LD in Europe and in North America (U.S. and Canada) are similar in clinical features but differ due to the variety of genospecies that cause the disease in Europe (*Borrelia afzelii, Borrelia garinii and Borrelia spielmanii*). Clinical criteria for case definition were not changed; however, laboratory confirmation is improved to require isolation of the pathogen’s bacterial culture from a clinical specimen or demonstrate diagnostic levels of IgM or IgG antibodies to the pathogen [28]. Because of the nonspecific nature of many clinical manifestations, laboratory (serologic) testing is essential. Epidemiologic findings about the likelihood of exposure to infected ticks inform the serologic testing. The results of laboratory tests, along with acceptable specificity and sensitivity of the tests, provide supportive evidence of infection. A negative test result could rule out the disease despite obvious symptoms and treatment would be withheld. This could affect reporting of confirmed cases and result in fluctuations in case counts. Canada, U.S. and European countries advocate a two-tiered serologic testing including enzyme-linked immunosorbent assay followed by Western blot which improves the specificity. Serologic testing is sensitive in very early stages of LD but two-tiered serologic testing is much more sensitive in disseminated LD, where the bacteria that cause LD have spread throughout the body [28]. Technical problems such as adoption of inadequate cutoff levels, the presence of cross-reacting antibodies, and false positive reactions caused by some autoimmune diseases could contribute to false-negative or false positive results [29]. Improvement of the diagnostic methods such as criteria used for test specificity and sensitivity and interpretation of the test for lab confirmation are important milestones for clinical diagnosis of LD.

The rise of annual reported human cases of LD argues for a better understanding of its geographic scope. Targeted surveillance for ticks and the infectious agent is needed to identify endemic locations as this knowledge is important in case definition, assisting clinical diagnosis and vaccine deployment. Studying the ecology of the disease [2] is very important because there are regional variations in the disease cycle. Identifying the individual drivers in different locations might aid in implementing vector and reservoir targeted interventions as well as prevention and control recommendations.

To our knowledge, this is the first comprehensive review on spatiotemporal patterns of LD and the use of geospatial technologies to study interfacing populations of the reservoir hosts, vectors and humans. While there is extensive literature which details the risk factors for LD and spatiotemporal factors affecting ticks, the infectious agent, host species, habitat and human exposure, a comprehensive review of GIS-based studies has not been conducted. This paper collates previous and current GIS research to define methods currently employed that have been the most effective in examining the spatial epidemiology of LD. Another goal is to determine additional methods that have not been employed in GIS-based LD research but have been used to study other tick-borne diseases that occur in conjunction with LD so as to also provide public health practitioners and policy makers with the spatial tools for use in vector and reservoir targeted strategies against Lyme disease.

### Recent Reviews

According to the review of Lyme disease epidemiology by Gerstenblith *et al.* [32], a myriad of studies into symptomology, causes, and treatment of LD exist. However, there is only one review on spatial aspects of LD transmission [33]. Killilea *et al.* focused on determinants of spatial variation in human incidence rates, tick densities and tick infection prevalence at multiple spatial scales [33]. They found that soil type, vegetation type and local host community affect vectors, hosts, and pathogens and their interactions at finer-scale. At the meso-scale, landscape composition, configuration and regional host community were found to play important roles. At coarser scales, macroclimate and biogeography were found to be the determinant factors. They suggested future research could focus on testing the effect of the same environmental variables at multiple scales. They found that long term data collection could provide wide coverage of variable biotic and abiotic conditions and consequently contribute to the progress made in identifying the determinants of spatial variation in LD risk. They suggested more standardized data collection and analysis methods given the current limitations of data collection and inconsistent methods of tracking human cases. The only environmental variable they found consistent with increased LD risk and incidence was the presence of forests. The percentage of land-cover edge consisting of adjacent forest and herbaceous land-cover was a significant factor where humans frequently come in contact with infected vectors. Factors effecting human behaviors that influence contact rates are least well understood and need to be explored.

Carver *et al.* has included a section on LD and other tick-borne diseases in their review of the ecology and epidemiology of directly transmitted and vector borne diseases [34]. The review concluded that most studies are short term and use season-specific climatic variables predicting temporal changes in tick abundance and LD risk [35]. They suggested that with the use of tick population monitoring programs over sufficient periods, a comparison of the fit of different climatic models to the data could be better evaluated. The authors emphasized long-term monitoring of the tick populations that are infected with zoonotic pathogens, and their most efficient reservoir host, the white-footed mouse. The abundance of mice is highly predictable in association with acorn production by oaks [36].

Donohoe *et al.* reviewed the literature concerned with LD and human risk factors [37]. They concluded that there needs to be more research done on translating science into real world solutions. In order to better understand the spatial reach of LD and risk maps, the tourism industry should be involved as a stakeholder and take an active role in disease surveillance by monitoring and reporting ground conditions at the tourism destinations. More research is needed to be done on activity-based risk and perceptions of risk and known factors and their influence on individual’s choice to engage in protective behavior. Research on the impact of LD upon the tourism industry needs to be considered in terms of an employee health, travel choices and the economic sustainability of tourism in LD-endemic areas.

Ogden *et al.* published a review in 2009 on the emergence of Lyme disease in Canada [38]. Their review concluded that Lyme disease is emerging in Canada because the range of *I. scapularis* is expanding in the eastern and central provinces. They found that warmer temperatures and dispersion of the ticks by migratory birds on animal hosts are the main factors for these vector establishments in Canada. Field studies validate the fact that risk maps for the geographic occurrence of *I. scapularis* are useful to identify areas where ticks are becoming established.

## 2. Methods

A literature search was conducted to identify recent articles discussing LD and the use of GIS and risk modeling. Several online databases were queried, including Google Scholar, Journal Storage (JSTOR), Cumulative Index of Nursing and Allied Health Literature (CINAHL), Web of Science, ScienceDirect, and Public/Publisher Medline (Pubmed). The following key words were used individually and in combination as inclusion criteria for articles to be considered for this review; LD, tick abundance, spatial distribution of LD, geographic information systems. Our review covers a 25-year period, inclusive of GIS-based studies published since 1990; 1990 was chosen as the starting point because the CDC recognized LD as a nationally notifiable disease and developed a national case definition in 1991. Initial searches yielded approximately 27 results. The abstracts of these publications were reviewed to confirm applicability. Studies that have used GIS and RS methods in the exploration of LD epidemiology were selected. After considering additional exclusion criteria (manuscripts with no GIS use, non-English language, manuscripts not available as full-text), 22 publications remained. Articles were summarized and grouped into six categories: Geographic distribution of ticks, reservoir hosts and pathogens; trends in surveillance; prevention and human behavior risk; climate change; population genetic analysis; and host-pathogen relationship. Table 1 presents these studies in detail under each category with GIS methods applied, study region and date, data, host, vector, pathogens and common risk factors. The articles categorized in Table 1 under Geographic distribution of ticks, reservoir hosts and pathogens; trends in surveillance; prevention and human behavior risk were summarized under *The Causal Explanation of LD Trends* in the Results section.

## 3. Results

The most compelling result arising from the published studies on exploring the ecology of Lyme disease is its still being on the Public health research agenda and fairly intensively studied. Exciting things are taking place in terms of proactive vector surveillance, prevention and control programs. More tick collection studies undertaken in response to the spatial expansion of Lyme disease. More studies are conducted on patterns of global climate change and their possible impacts on Lyme disease. Characteristics of these studies are summarized in Table 1.

Most studies attempt to quantify the associations between LD risk variables (e.g., vector, pathogen, and host abundance and distribution) and environmental variables using the spatial analysis capabilities of the GIS. Because ticks have limited mobility, their reservoir host range is an important spatial determinant. Long term field monitoring of the host-seeking ticks in the host range help define landscape predictors of LD risk. In places where field data are not available, tick distribution is predicted based on *a priori* information about tick species ecology. RS/GIS are powerful tools for enabling the prediction of LD risk. Ticks’ dependence on certain environmental factors (*i.e.*, climatic factors) are experimentally-verified through *a priori* approach. Predictive maps of tick distributions are also produced by *ad hoc* statistical models (e.g., regression, discriminant analysis, *etc.*), based on GIS-based data reflecting the relationship between occurrence and a number of spatial covariates (*i.e.*, vegetation, climatic, geological, and hydrological, soil types, host population covariates). Spatial scale of the study matters in facilitating characterization of the landscape in terms of vector and pathogen prevalence. Field data are usually collected over small areas; it is difficult to scale up these observations to longer time periods or larger spatial scales. Pepin *et al.* should be complemented on conducting a large scale study including 36 eastern states in the United States [5]. Pepin *et al.* found that the relationship of daily nymphal densities and human incidence was strongest in high-prevalence areas, and varied by region and state due to the distribution of *Borrelia burgdorferi*. They compared model-based predictions of indices with raw density indices. They integrated GPS based field data into GIS-based density mapping and predictive models. Their study area included 2411 counties in 36 states at 8 km × 8 km spatial scale. The estimated tick density indices explained the variation of incidence better than the raw data because they account for effects of other factors such as landscape fragmentations, spatial autocorrelation and weather that play key roles on tick and pathogen distributions. Their findings verify that modeling risk based on habitat alone without follow up data on the distribution of human cases and vectors could be misleading. The adequacy of the environmental indices has to be tested by comparing the geographic distribution of risk areas with both the distribution of vectors or human cases, and preferably both.

Vector establishment is an important factor for vector surveillance. One stage of the tick found at one site could indicate recent introduction or poor micro-environment Finding all three stages of the tick could indicate a population has been established [39]. Assessing potential non-endemic areas with the necessary combination of environmental factors for established populations is a novel approach. Guerra *et al.* applied this approach and studied the distribution of *I. scapularis* in the upper Midwest and environmental factors affecting its establishment [40]. They adopted methodologies from macro-level studies in Europe [22,41,42,43] and in the United States and Canada [44,45,46], which used satellite, climatological and ecologic data to determine the vector tick habitats. Their GIS-based environmental model was based on the hypothesis that tick abundance is an indicator of the suitability of environmental conditions for reproduction and survival. Soil order and land cover were the dominant contributors to tick presence. They constructed risk maps indicating suitable habitats within areas where *I. scapularis* is already established.

**Table 1:** Summary of studies with common risk factors.

GIS Analysis/Citation	Region/Date	Host, Vector, Pathogen	Data	Common Risk Factors
***Geographic distribution***				
GPS based Field data integrated into GIS, [5]. Zonal Statistics Spatial Autocorrelation, Predictive modelling Density mapping	36 Eastern States 2004–2006	*Ixodes scapularis*	County level Human Case reports to the CDC as part of the national Notifiable Disease Surveillance System (NNDSS) Field-derived tick data. Monthly vapor pressure, maximum daily temperature. Normalized vegetation index (NASA) Elevation (USGS National land cover database)	The distribution of *B. Burgdorferi* genotypes. The estimated density of infected nymphs
Risk mapping, [40] GPS based Field data integrated into GIS, habitat analysis	Wisconsin, Illinois, Michigan 1996–1998	*Ixodes scapularis* white-footed mice, chipmunks	Field-derived tick data. Vertebrate collection. Soil samples collection(top soil, leaf litter thickness, slope, pH, soil texture). Field-derived Forest Moisture Index Climate data from NOAA (yearly and seasonal precipitation). Bedrock geology USDA Forest Service	Tick presence positively associated with deciduous, dry to mesic forests, and alfisol types of soils with loam-sand textures. Tick absence associated with grasslands, conifer forests, et/wet mesic forests, acidic soils of low fertility and a clay soil texture, Precambrian bedrock
Geostatistics [47] Spatial autocorrelation	Rhode Island	*Ixodes scapularis*	State-wide collected human incidence data	A highly significant spatial trend for decreasing number of ticks and incident cases of LD with increasing latitude. Exposure to deer ticks and LD risk occurs mostly in the peridomestic environment.
Space-time scan statistics [48]	Virginia 1998–2011	N/A	Census tract level count of LD human cases	Spatial expansion towards south and west along eastern coast of the U.S. Areas where education and surveilliance needs are the highest
GPS based Field data integrated into GIS, Density mapping, [49]	New York	Deer, mice, chip munks	Growing season temperature, precipitation, abundance of hosts and acorns	Risk associated with prior year’s abundance of mice and chipmunks and acorns
Density surface mapping [50] RS techniques Supervised Classification	California Mendocino County	*I.Pacificus*	Field-dervied data: tree species, deer signs, NDVI, sunlight, hydologic data	GIS-based environmental data could predict nymphal density more accurately than field-derived data
Density surface mapping [51] Habitat analysis	California Mendocino County	*I.Pacificus*	Climatic variables, habitat type, deer usage on tick-related traits	A shift from peak nymphal densities occuring in oak woodlands in spring to redwood habitats in summer
Clustering [52] Habitat analysis	Middle Atlantic region of U.S. 1997–1998	*I.Scapularis*	Land cover, distance to water, forest edge, elevation and soil type	Clustered pattern along coastal plain of the Chesapeake Bay
Spatial heterogeneity [53,54,55] Spatial autocorrelation Accuracy assessment			Species-habitat relationships. Species-environment relationships	Spatial autocorrelation improves predictive spatial models
***Trends in Surveillance***				
Proximity Analysis, [56] Geographicstratification techniques	Czech republic 1997–2010	*Ixodes ricinus*	Human Population Migration and Demographic changes from Czech statistical office LD human cases	Population density, high incidence among 50–65 years old people and 10 years old children. Socio-economic transformation. Amount of time people spent outdoors Peri-residential distribution and home vicinity
Clustering analysis [22] Population density analysis	Germany 2009–2012	*Ixodes ricinus*	Notified cases of LD Clinical LD manifestations	Urban areas, Forested areas and public parks. Free-range livestock husbandry
Clustering analysis [57]	Belgium 1994–2004	*Ixodes ricinus*	Deer population density Land Use, Forest cover	Human incidence, Roe deer population, Forest cover, Population density, peri-urban areas
***Prevention behaviuor risk***				
Surveys [58]	New York	N/A	Voluntary, anonymous questionnaire.	Participants having a family member with LD were more likely to use preventive behaviors
Cross-sectional [59]. Logistic regression	New Jersey 1988	*Ixodes scapularis*	Occupation	Outdoor work
Geographic Stratification techniques [60]	Missouri	*Ixodes scapularis*	Structured interview Park types	Human Population density estimates
***Climate Change***				
A Review of expert workshops, Multivariate analyses and predictions [61] Wavelet-based time series analysis [62]. The Normalized difference water index (NDWI). Cluster Analysis of high disease risk. Based on human cases	Belgium 2000–2010	*Ixodes ricinus*	Incidence, prevalance, distribution of infections through various routes (vector, rodent, water, food, air) NDWI CORINE land cover map obtained from European. Environment Agency MODIS data (Moderate Resolution Imaging Spectroradiometer) obtained from Land Processes Distributed Active Archive Center	Proposed to build an integrated network for environmental and epidemiologic data LD incidence. Vegetation greenness and moisture. Local characteristics of vegetative systems. Multiresolution analysis. Lagged climatic effects, vegetation and moisture related events spanning periods of 2 or more years
Wavelet-based time series analysis [63]. The Normalized difference water index (NDWI). Spatial autocorrelation Voronoi polygons. Cluster Analysis of high disease risk	Belgium 2003–2010	*Ixodes ricinus*	GDD (growing degree days) values calculated for each season derived from hourly temperature data from National Climatic Data Center and Royal Meteorological Institute of Belgium	Vegetated areas and frequent weather anomalies. GDD (Growing Degree Days) indicator of heat accumulation. Seasonal conditions affect the incidence
Global Climate Modelling [64] for two greenhouse gas emissions	Canada 1970–2000	*I. Scapularis*	Grid point data of projected daily maximum and Minimum temperatures obtained from two models: CGCM2 (Coupled Global Climate Modelling and Analysis) UK Hadley Center’s HadCM3 model	Annual degree days (DD > 0 °C), seasonally variable temperature conditions. A2: Increasing heterogeneous population, fragmented economy, technology change; B2: Intermediate levels of economic growth and lower population growth
Global Climate Modelling [65] for two greenhouse gas emissions	Canada 2020s, 2050s, 2080s	*I. Scapularis*	Minimum temperatures obtained from two models: CGCM2 (Coupled Global Climate Modelling and Analysis)	Annual degree days (DD > 0 °C), seasonally variable temperature conditions. A2: Range moved northwards by 200 km by the 2020s and 1000 km by the 2080s; B2: Projected expansion between 2050s and 2080s
Risk Mapping [1] Simulation Models. Validation through field studies	Canada 1970–2000 Projected 2020s, 2050s, 2080s	*I. Scapularis*	Field-derived tick and rodent data. Vertebrate collection. Human Population data at census-sub division level obtained from Statistics Canada. Index numbers of ticks migrating on migratory birds. Percentage cover of forest habitat	Vector populations, ambient temperature, number of nymphal ticks immigrating on migratory birds and forest habitat cover. Predicted temperature conditions and emission scenarios
***Population genetic analysis***				
GPS and field mapping [66]. Spatial expansion mapping [67]	Virginia 2011	*I. Scapularis*	Field derived tick data. Molecular methods	Population genetic signals of nymphal *I. Scapularis.* Eastern most ticks with demographic expansion but not spatial expansion. Central and western tick populations with spatial expansion
***Host-pathogen relationship***				
Range expansion mapping [68]. Spatial structuring	European strains. Chinese strains	*B. garinni.* *B. afzelii*	Multilocus sequence analysis. Historical populations of *B. garinni and B. afzelii*	Geographic distances between collection sites. Rodent population expansions after the glacial maximum
Prevalence mapping of antibodies [69]. Clustering	Northeast, Upper Midwest. West Coast, U.S.	*B. burgdorferi*	County residence of each dog tested by zip code. County level population data by U.S. census	Antibodies to *Borrelia* in dogs by zip code
Finer scale prevalence mapping [70]	California	*B. burgdorferi*	CALVEG 2000 (vegetation coverage obtained from California Forestry and Fire protection). Precipitation isohyetal polygons	Seropositive and seronegative coyote locations, Vegetation cover and rainfall
***Vaccine deployment***				
GPS and field mapping [17]. Nymphal infection prevalance	New York	*B. burgdorferi*	Field derived tick data	Significant decreases in tick infection prevalence were observed within 3 years of vaccine deployment.

Nicholson and Mather even furthered this approach and examined the association among LD in humans and the degree (engorgement) of nymphal blacklegged tick, *Ixodes scapularis.* They demonstrated that exposure to deer ticks and LD risk occurs mostly in the peri-domestic environment in Rhode Island [47]. They found a highly significant spatial trend for decreasing number of ticks and incident cases of LD with increasing latitude. They incorporated geostatistics (e.g., Kriging), to model spatial autocorrelation of tick densities. These findings were combined to create a model that predicted LD transmission risk.

Most of these studies look at the spatial expansion of LD human cases to determine the areas where vector surveillance is most needed. There is no standardized approach that considers where and when vector surveillance should be done. Li *et al.* applied space and space–time scan statistics to reveal the spatial and spatio-temporal clusters of LD [48]. They used finer scale census tract level count data of LD human cases in Virginia from 1998 to 2011. Their findings confirmed a spatial expansion towards the south and west in states along the eastern coast of the United States. They concluded that these are the areas where education and surveillance needs are highest.

Temporal dynamics of reservoir host’s food and shelter conditions play a key role in tick distribution. Ostfeld *et al.* researched the times of highest entomological risk for LD. They found the strongest predictors of the risk for LD was based upon the abundance of mice and chipmunks in the previous year, and the abundance of acorns from the previous 2 years. They concluded that prior abundance of key hosts for the immature stages of the tick vector and existence of critical food resources for those hosts determine the inter-annual variation in entomological risk of exposure to LD [49]. They tested the significance of ambient growing-season temperature, precipitation, two indices of deer (*Odocoileus virginianus*) abundance, and densities of white-footed mice (*Peromyscus leucopus*), eastern chipmunks (*Tamias striatus*), and acorns (*Quercus* spp.), in predicting LD risk. Their data were derived from 13 years of evaluation of several field plots within eastern deciduous forests of New York State in the epicenter of U.S. LD.

For vector surveillance, recent studies have applied integration approaches that combine spatial modeling using GIS and Remote Sensing with follow-up field-derived data models. RS enables identification of tick habitats from multispectral images. The increasing supply of RS data have led to wider applications of habitat modeling, and predictive mapping. Remotely sensed measurements from satellites can provide large scale data with good spatial and temporal resolution, but they require validation on the ground. Supervised and unsupervised classification of tick habitats is more about the structural characteristics of vegetation (distribution of vegetation biomass horizontally and vertically) rather than ground-based characterization (e.g., canopy cover, *etc.*). Studies first model the host/vector behavior in relation to environmental and climatic conditions and project/predict potential tick distributions under current climatic conditions. Then, the accuracy of GIS/RS-based modeling are validated with field–derived data models [49,50,51]. Eisen *et al.* mapped the high-risk areas of human exposure to LD by creating a continuous nymphal density surface for the entirety of Mendocino County in northwestern California in 2004 through habitat classification and GIS/RS models [50]. The resultant surface showed that 11.9% of the county was classified as habitat posing at least moderate risk of human exposure to nymphs (>6.4 nymphs per 100 m^2^). They found high-risk clusters in the central interior and most heavily populated region of the county, whereas low-risk areas were in close proximity to coastal population centers. They used a supervised classification model (uses *a priori* knowledge of the land-cover classes), based on multi-seasonal Landsat TM 5 images, to identify the key habitat of *I. pacificus* nymphs. They determined the density of nymphs in 62 woodland-leaf areas located throughout Mendocino County and explained the variation in nymphal density based on field-derived data and observations (e.g., tree species present, deer signs, or using GIS-based environmental data). Using July NDVI (a remotely sensed vegetation index of plant “greenness”), November greenness, a coastal influence category, May solar insolation, November hours of sunlight, and dominant hydrologic grouping as input variables, they could predict nymphal density 22% more accurately at 16 validation sites (*r*^2^ = 0.72) than their field-derived data model (*r*^2^ = 0.50).

Two studies by Eisen *et al.* [51] and Bunnel *et al.* [52] examined the temporal (seasonal) patterns and the spatial extent of variation in peak and cumulative densities of infected *Ixodes pacificus* nymphs. Eisen *et al.* examined environmental characteristics such as climatologic variables, habitat type, deer usage on the tick-related traits within the Mendocino County in California [51]. They found the average durations of total and peak nymphal density were 31% and 82% longer, respectively, in areas with conifers present than in oak woodlands, which represented the warmest and driest habitat type. Their results revealed a shift from peak nymphal densities occurring in oak woodlands in spring to redwood/tanoak habitats in summer.

Bunnel *et al.* [52] studied abundance patterns of the black-legged tick, *Ixodes scapularis* in the Middle Atlantic region of the U.S. In 1997 and 1998, 663 adult *I. scapularis* ticks were collected from 320 transects spanning 66,400 km^2^ in five states of the Middle Atlantic region. They found clustered patterns, with relatively high numbers along the coastal plain of the Chesapeake Bay, decreasing to the west and south. There were significant associations between tick abundance and land cover, distance to water, distance to forest edge, elevation, and soil type.

These studies found clustered patterns, but did not attempt to address the possibility of spatial heterogeneity (the potential for environmental relationships to vary spatially) in species-habitat relationships. The reservoir host-tick-human interaction in different environments cause a great deal of variation in the distribution of LD within endemic zones. Wimberley *et al.*, however, addressed the possibility of spatial heterogeneity (the potential for environmental relationships to vary spatially) in species–habitat relationships by delineating geographical zones with similar species-environment relationships, which can then be used to stratify landscapes for the purposes of further predictive modelling [53]. By incorporating spatial autocorrelation (the tendency for distributions to be clustered in space) or spatial heterogeneity into predictive spatial models, environmental predictions of the geographic distribution of reservoir hosts and vectors could be improved [54]. In a comparative study, Wimberley *et al.* assessed the accuracy that is gained by applying spatial autocorrelation and heterogeneity in LD risk mapping [55].

### 3.1. The Causal Explanation of LD Trends

The causal explanations of LD trends in North America varies based on study scale. The current endemic areas of LD in North America at the regional level are northeastern United States, Southeastern Canada, upper Midwest and coastal central and northern California. Counties with less fragmented forest cover has shown strong positive relationship between human incidence and tick densities, but urban counties with dispersed forests did not show that strong positive relationship [5]. In California, at the county level analysis, high-risk clusters were located in the central interior and most heavily populated regions, whereas low-risk areas were in close proximity to coastal population centers [51]. Finer scale analysis at the zip code level revealed isolated clusters of elevated LD incidence in close proximity to forested areas in California [51]. Based on a finer scale analysis at the census tract level, states along the eastern coast of the United States [36] and coastal plains had higher incidence with increasing latitude in peri-domestic (peri-urban) environments [52]. These result validates that high resolution incidence data is an important attribute of LD research because it captures isolated endemic areas.

As in the case of North America, geographic scale also mattered in the causal explanation of LD trends in Europe. LD infection rates increased by settlement size. Peri-urban areas with isolated houses and forests contributed to the increasing trends in many parts of Europe. A recent Czech study by Zeman and Benes [56] found that this trend is driven by increasing infections acquired at or nearby a residence, and a noticeable extension of the prevalence season from earlier in spring to later in autumn. They classified cases as “local” or “remote” depending on whether distance between residence and place of infection exceeded a 5 km limit. Municipalities were classified by size according to the number of residents registered during the study period. Local infections increased in the smallest (less than 500 inhabitants) and mid-sized (2500 to 25,000) settlements, in contrast to largest settlements of 50,000 inhabitants. This shows that the growth is progressive in expanding settlements compared to those experiencing population declines (all age cohorts, inclusive of small children and the oldest seniors migrating predominantly out of cities). These peri-urban locations have intense residential construction with new dwellings erected every day as a result of the liberalized housing and real estate market after the political and economic transformations that have taken place in the country. This process led to increased contact between the populations and the tick habitats. The amount of time people spent outdoors around their homes has increased not only due to adopting rural manor houses but also lifestyle changes.

Wilking and Stark [22] analyzed geographic patterns and time trends of notified LD cases in Germany. They discovered that human infection was not restricted to forested areas but also occurred in urban areas with public parks such as those in Frankfurt and Schwerin. Small-scale mapping of cases in Berlin showed peripheral neighborhoods were less affected. LD incidence revealed a pronounced seasonality starting in April, reaching the peak in July and declining in December. Urban counties exhibited the highest incidence during the period of 2009 and 2012. The incidence was highest in the cities of Frankfurt and Schwerin and lowest in Berlin.

LD infections exhibited a large linear increase in Belgium over the last decade. Linard *et al.* studied the spatial distribution of LD in Belgium [57]. They found that the spatial distribution of LD is highly clustered along a North-South axis, in the provinces of Antwerp, Brabant and Namur. LD patients were georeferenced to the municipality where infection took place. They assumed the municipality of infection that was recorded was the residence. They combined socio-economic and environmental characteristics of municipalities in explaining the disease occurrence. The population density and the proportion of developed areas determined the urbanization of municipalities. Explanatory variables that were provided as input into their spatial model included host population (roe deer) and forest cover, and the human population living in separated houses and the socio-professional level of the population sampling. Their findings revealed that LD is associated with recreational and peri-domestic outdoor activities in high income, peri-urban areas with isolated houses and forests.

The causal explanation of LD trends were examined also by studies examining the behavioral risk of exposure to tick-borne diseases focusing on regions endemic for LD [58], and among individuals with occupational exposure [59]. Bayles *et al.* measured the preventive behaviors of visitors to recreational parks in the St. Louis, MO area, an endemic area to tick borne diseases other than LD [60]. They used geographic stratification techniques. They created 5 km radius buffers around the perimeter of each site, and overlaid the buffers on a map of census blocks with population estimates from the 2010 U.S. Census. Based on human population densities, they classified parks as either suburban, exurban or rural. Results presented significant differences in behaviors across parks. Those in exurban parks were more likely to perform frequent tick checks and use insect repellents, while those in suburban parks were more likely to avoid tick habitats. On the other hand, those in rural parks were less likely to avoid tick habitats.

### 3.2. Climate Change

Climate change is an important factor in the spread of LD because areas that are not currently endemic may become so due to climate change. Climate may induce alterations in the spatial distribution of LD and temporal LD incidence [61]. LD is a sensitive disease to climate change. Warming, humidity and moisture are important factors of the biology of tick survival and dispersion. Climate change can directly affect LD transmission by affecting ticks’ geographic range, increasing rates of reproduction, affecting biting behavior, and shortening incubation periods of the pathogen. These hypothesized effects have been tested by a number of studies that examined climate change through climate change models, remotely sensed data and GIS. For example, Barrios *et al.* used vegetation greenness and moisture—the Normalized Difference Water Index (NDWI) as an explanatory variable in the modeling of LD incidence in Belgium [62]. NDWI was selected as an indicator of vegetation moisture because it expresses the contrast between the sensed reflectance in the short wave infrared (SWIR), which diminishes as vegetation water content increases. Humidity and soil moisture are important factors of tick ecology. They used Moderate Resolution Imaging Spectroradiometer Sensor (MODIS) during the periods 2000–2010 and focused on Neufchateau and Turnhout districts with epidemiological relevance. Wavelet-based time series analysis was employed to assess timing connections between the events occurring in the habitat and disease incidence. The vegetation and moisture related events spanning periods of 2 or more years were influential in shaping the temporal LD incidence. A reduction of LD incidence was noticed in southern Belgium as a consequence of the 2003 drought.

Barrios *et al.* further examined the links between climate, vegetation and LD by monitoring vegetated areas and frequent weather anomalies in a subsequent study [63]. LD has been responsible for major outbreaks in Belgium in the last decade and its risk could increase with climate change. Growing degree days (GDD) values were derived from hourly temperature data and calculated separately for spring, summer and autumn from Royal Meteorological Institute of Belgium. GDD represents the cumulative effect of heat units and has been used for assessing the timing of various biological processes. During their study period of 2003–2010, they assessed the temporal variability of LD risk by computing vegetation indices from MODIS images and combining with meteorological data. Their risk maps showed existence of spatial autocorrelation in disease risk and exhibited cluster size changes. Two resultant northern and southern zones presented differences in their landscape composition.

Three articles by Ogden *et al.* focused on potentially how far *I. scapularis* extend north into Canada with climate change scenarios. Ogden *et al.* [64] mapped the geographic limits for *I*. *scapularis* in Canada using annual degree days (DD > 0 °C), a good indicator of the seasonally variable temperature conditions. They used two Global Climate Models; the Canadian CGCM2 and the UK HadCM3 for two greenhouse gas emission scenario enforcements, “A2” and “B2”, defined by the Intergovernmental Panel on Climate Change [65]. In scenario A2 the future world has increasing heterogeneous population, fragmented economic growth and technology change. In scenario B2, the future world has intermediate levels of economic development and a lower population growth rate than A2.

In a follow up study, Ogden *et al.* [66] examined how the geographic limits for *I*. *scapularis* establishment may change with climate change scenarios for three future projections: 2020s, the 2050s and 2080s. They found that using either climate model, under scenario A2, the theoretical range of *I.* *scapularis* has extended northwards by approximately 200 km by the 2020s, and 1000 km by the 2080s. Under scenario B2 reductions in emissions had little effect on projected range expansion up to the 2050s. The projected expansion between 2050s and 2080s was less than that under scenario A2.

Ogden *et al.* [1] developed risk maps for the occurrence of the LD vector *I.* *scapularis* in Canada under current and projected climate [1]. In order to calculate algorithms to estimate risk, they combined simulation models of vector populations, ambient temperature, number of nymphal ticks immigrating on migratory birds and forest habitat coverage. They mapped low, moderate and high risk regions from 1970–2000 temperature normals to the 2080s using predicted temperature conditions and emission scenarios. Risk maps were validated with field studies under current climate conditions. An index of certainty of ticks was determined for each field site. Their novel approach using information on the biology of vector survival and dispersion helps assess the spatial and temporal risks for vector borne diseases that are not currently endemic but may become so due to climate change.

### 3.3. Population Genetic Analysis

Studies of phylogeographic analysis yields valuable insights into LD evolution/emergence and seeks answers to the following questions: Does LD vary genetically over geographic and temporal scales? Is transmission distance-related and varied seasonally in response to finer-scale microclimatic and landscape characteristics? Two studies could identify signals of spatial and demographic tick populations through population genetic analysis. Kelly *et al.* [67] studied the demographic and spatial expansion of *I. scapularis* in Virginia through examining population genetic structure. They found strong signals of both demographic and spatial expansion in Virginia and attributed that this expansion may count for dramatic rise in human LD in the state. Eastern-most tick populations showed demographic expansion but not spatial expansion in contrary to central and western tick populations. This could be due to drops from migratory birds or imported from deer at nearby coastal locations. Inability of detecting differences in genetic diversity among sites could suggest that ticks may not be dispersal-limited. There is potential for differences in host preference and questing behavior among different *I. scapularis* populations in Virginia. They are likely to have originated in the South and then radiated upon populating northern forests following the retreat of glaciers [68].

### 3.4. Host-Pathogen Relationship

Host-pathogen relationship is an important relationship to study in LD epidemiology because LD could involve the same agent but different ticks and hosts in different regions. This could lead to regional variations in the LD transmission cycle. LD cycle could be maintained in wildlife but might not transmit to humans because of the biting preference of certain ticks. Vollmer *et al.* [69] studied the level of geographic structuring of populations of LD group species (*burgdorferi* species group) to determine if they are consistent with patterns of dispersal of their different vertebrate host groups. They specifically studied the bird-associated species *B. garinii* and rodent associated *B. afzelii* populations. They analyzed *Borrelia* isolates from England, Scotland, Latvia, Germany, Switzerland, France and China, and compared European strains to Chinese strains. They plotted rank pairwise genetic distance between strains against the rank geographic distance between collection sites using Spearman’s rank correlation. The European correlations were found to be significant. Results demonstrated range expansions of *B. garinii* and *B. afzelii* populations in Europe in the distant past. In response, they proposed that the expansion of *B. afzelii* in Europe might be linked to rodent population expansions after the last glacial maximum. Strains have expanded and spread out of a refuge in the distant past with their rodent hosts.

Bowman *et al.* studied LD caused by infection with the *Borrelia burgdorferi* in dogs [70]. They mapped antibodies to *Borrelia* in dogs by aggregating each dog tested to postal zip code into counties and states in the United States. Percent positive test results were calculated by dividing the number of dogs reported positive for *Borrelia burgdorferi* by the total number of dogs tested. The prevalence map showed the most likely clusters in the coastal Northeast, upper Midwest and West Coast. Hyper-endemic areas with positive rates greater than 40% were clustered in Northeast and Midwest.

Foley *et al.* examined spatial and temporal relationships among *B. burgdorferi*-exposed coyotes with vegetation type and climate in California [71]. Coyotes are well distributed in various ecosystems in California and inhabit both peri-domestic and wild land. They looked for associations of seropositive and seronegative coyote locations with vegetation cover and precipitation layers in the GIS environment. Their spatial analysis showed a non-random pattern of seropositive coyotes with increased seropositivity in blue-oak foothill pine, montane hardwood and redwood vegetation regions, and decreased seropositivity in coastal sagebrush and cropland. Increased rainfall was associated with higher seropositivity of *B. burgdorferi.* They found increased exposure to *B. burgdorferi* in blue oak woodlands. The findings of this finer scale study provided valuable insight on host-pathogen relationship and tick-to-human transmission at local ecological processes.

### 3.5. Vaccine Deployment

Breaking the LD transmission cycle starts in the wild by targeting the reservoir host. There are very few published accounts of GIS and GPS utilization in host-targeted methodologies being detailed for vaccine deployment in LD epidemiology. Transmission risk declines with increasing distance between susceptible host and the pathogen source. Understanding current conditions of spatial interaction between hosts and vectors and the spatial distribution of these organisms would help predict future distributions and facilitate better vaccine deployment. Simple applications might involve determining the location of sampling sites, plotting maps for use in the field studies. More complex applications take advantage of GIS-based suitability modelling to determine the best locations for bait stations containing a pesticide-delivery system. A study by Richer *et al.* found that significant decreases in tick infection prevalence were observed within 3 years of vaccine deployment [17]. In a prospective 5-year field trial, they showed that oral vaccination of wild white-footed mice resulted in outer surface protein A-specific seropositivity that led to reductions of 23% and 76% in the nymphal infection prevalence in a cumulative, time-dependent manner (2 and 5 years, respectively), whereas the proportion of infected ticks recovered from control plots varied randomly over time. Significant decreases in tick infection prevalence were observed within 3 years of vaccine deployment. Their results suggest that implementation of such a long-term public health measure could substantially reduce the risk of human exposure to Lyme disease.

## 4. Conclusions

Several conclusions emerged from this review. First of all, understanding Lyme disease starts with identifying its spatial characteristics. Spatial analysis of LD contributes to epidemiologic knowledge of exposure to infected ticks which informs diagnostic testing and assists clinicians in the accurate diagnosis of LD. Spatial analysis provides high levels of insight into understanding the conditions under which ticks spread, risk areas could be highlighted and environmental and climatic factors behind the prevalence of LD could be determined.

Specifically, the distribution of *B. Burgdorferi* genotypes, the density of infected nymphs [5] and the presence of forests [56] were found to be consistently associated with increased LD risk and incidence. Heterogeneous landscapes with fragmented land use mixing forests and houses are associated with higher risk and incidence than other landscapes [56]. Some forests exhibit higher LD risk and incidence than others. For example, tick presence is positively associated with deciduous, dry to mesic forests. Contrary, tick absence associated with grasslands, conifer forests, wet/wet mesic forests [40].

Alfisol types of soils (semi-arid to humid areas, typically under a hardwood forest cover) with loam-sand texture had a positive correlation with tick presence. Tick absence was associated with acidic soils of low fertility and a clay soil texture [40].

Some findings were related to seasonal variations such as a shift is observed from peak nymphal densities occurring in oak woodlands in spring to redwood habitats in summer in California [51]. LD Risk is associated with prior year’s abundance of mice and chipmunks and acorns in eastern United States [49].

Lagged climatic effects, vegetation and moisture related events spanning periods of 2 or more years had an impact on LD risk [62]. Local characteristics of vegetative systems such as vegetation greenness and moisture, frequent weather anomalies, seasonally variable temperature conditions (Growing Degree Days; Annual Degree Days) had an association with higher incidence [63]. Vector populations, ambient temperature, number of nymphal ticks immigrating on migratory birds and forest habitat cover are associated with higher incidence in Canada [1].

Current LD research also focused on potentially how far ticks extend north into Canada with modelling climate change scenarios. Given the scenario of increasing heterogeneous population, fragmented economy, and technology change, there were observable and reported changes in the range of *I. scapularis* and LD cases in Canada moving northwards 200 km by the 2020s and 1000 km by the 2080s [64]. Given the scenario of intermediate levels of economic growth and lower population growth it is projected to expand between 2050s and 2080s [66]. These changes were due to an increase in temperature and seasonally variable temperature conditions. Warming climate trends affect ticks’ geographic range and increasing rates of reproduction [66]. Tick populations are highly sensitive to temperature at multiple life stages but nymphal stage is most relevant for transmission of agent to humans. The timing of nymphal host seeking is correlated with ambient temperatures and humidity. Warmer weather encourages outdoor activities and, therefore, the chance of encounters between infected ticks and humans increases.

Based on a study in Rhode Island [47], occurrence of plant communities (forests) suitable for sustaining vector populations was not predictive of Lyme disease risk. Instead, a highly significant spatial trend was observed for decreasing number of ticks and incident cases of Lyme disease with increasing latitude [47]. Population genetic analysis results confirm that in the United States, eastern most ticks exhibited demographic expansion but not spatial expansion but central and western tick populations exhibit spatial expansion [67,68]. Rodent population expansions after the glacial maximum had an impact on geographic distribution of LD incidence [69]. Vegetation cover and rainfall are associated with seropositive and seronegative coyote locations in California [71].

Exposure to deer ticks and LD risk occurs mostly in the peri-domestic (peri-urban) environment [40]. In Eastern Europe, countries (*i.e.*, Czech Republic) with political and economic transformations had LD risk in peri-residential areas by the vicinity of newly constructed homes [56]. High incidence was observed among 50 to 65 years old people and 10 years old children [56]. Amount of time spent outdoors in these new economies had an impact on LD incidence. Based on a US study, participants having a family member with LD were more likely to use preventive behaviors [58]. Outdoor work [59] and human population density estimates are associated with higher incidence [60]. In parts of Europe (*i.e.*, Germany), urban counties with forested areas and public parks exhibited highest incidence. In the United States, human incidence is higher in low—than in medium—density residential developments [2]. LD expands towards south and west along eastern coast of the U.S. There is a clustered pattern of LD incidence along coastal plain of the Chesapeake Bay. Rural landscapes are associated with higher incidence [52]. Areas with education and surveillance needs are the ones with the highest LD risk [48].

Another important conclusion was that Reservoir-targeted vaccine development appears effective in preventing tick transmission [72]. Sustained deployment of vaccines can decrease risk exposure to LD to all potential reservoir species over time. Implementation of a long-term vaccine development as a public health measure could substantially reduce the risk of human exposure to Lyme disease [17].

The breath of GIS applications and approaches applying spatial analysis techniques to LD epidemiology varied in the reviewed studies. One finding was very interesting that GIS-based environmental data could predict nymphal density more accurately than field-derived data [50]. Some studies were follow up field studies integrating GPS based field data into GIS [5,17,40,49,67]. Variety of data were mapped, such as tick densities, pathogen genotypes, human incidence and population demographics, host and vector habitats. Variety of mapping approaches were applied such as density surface (tick) mapping [5,49,50,51], risk mapping [1,40], population (human) density mapping [22], spatial expansion mapping of population (pathogen) genetic signals [68], range mapping of rodent populations and prevalence mapping of antibodies [68,71] and prevalence of nymphal infection [17]. Some studies used GIS to analyze and model habitats. GPS-based field data were integrated into a GIS for habitat analysis. They used land cover data, proximity measures (distance to water, forest edge), soil variables (soil types, soil texture, soil Ph), leaf litter thickness, slope and elevation data, and forest cover (tree species, forest types). Density surface mapping, zonal statistics, predictive modelling, and geostatistical tools were commonly used to perform spatial analysis [38,40,51,52]. Spatial variability in land cover, soils, and geology affect habitat suitability for vector species. Predictive modelling studies of tick and host distributions have applied spatial interpolation, spatial modeling and spatial clustering techniques based on environmental indicators. Spatial autocorrelation improved predictive spatial models [53,54,55]. Risk maps are developed by vegetation cover, moisture and temperature variables derived from satellite imagery.

To model and project the related potential alterations in climate change on the distribution of ticks, data sources from NASA, USGS, CDC, NOAA and (climatic and environmental data) are utilized in the development of spatial models. Monitoring tick abundance provides epidemiologically relevant information as well as tick absence so that statistical methods and predictive models can reveal the relationships of biological distributions with climate covariates. Maximum daily temperature, growing season temperature, annual degree days (DD > 0 °C), growing degree days, monthly vapor pressure (humidity measure), yearly and seasonal precipitation were the variables used to model and project the related potential alterations in climate change on the distribution of ticks. In order to test the hypotheses of the connections between climate and LD, wavelet-based time series analysis, and spectral indices, such as the Normalized Difference Vegetation Index (NDVI) and Normalized Difference Water Index (NDWI) are routinely used as remotely sensed measures of vegetation greenness and moisture. Spatial interpolation of climate data have been investigated as complementary approaches to predict spatial variations in frequent weather anomalies such as monthly climate.

There are a number of potential issues and limitations of spatial analysis on Lyme disease. Cases are reported on the basis of the patient’s residence rather than on the location in which the exposure occurred. Therefore, Lyme disease in a traveler returning from an area in which the disease is highly endemic cannot be construed as evidence of local transmission. Spatio-temporal component could provide misleading results because of the movement of the population between the time of infection and the onset of symptoms [73]. Inconsistent methods of tracking human cases (e.g., as case numbers rather than incidence or incidence rates) and incomplete disease reporting of confirmed cases could result in fluctuations in case counts and reported rates, which in many instances vary between provinces or states within a country. Over-reporting in non-endemic areas and under-reporting in endemic areas could cause spatially biased results [74]. More standardized data collection and analysis methods are needed given the current limitations of data collection and inconsistent tracking methods. National, provincial, and municipal boundaries are used for counts of human cases as part of notifiable disease surveillance systems for mapping human incidence data. These administrative boundaries are arbitrary boundaries that do not coincide with biologic boundaries. This presents a potential problem since biologic boundaries contain ecological conditions of habitats, which affect the distribution and density of vectors and host animals involved in the transmission.

One of the drawbacks of researching LD epidemiology is that there are still not good baseline data sets available on vector surveillance. Ground-verifying field studies are time-consuming and expensive. Empirical data on tick density and tick infection rates are difficult to collect for large areas. Therefore, there are very few large scale studies researching the geographical variation in the relationship between human case surveillance data and tick densities [5]. Region-specific standardized data collection and analysis approaches are needed to identify the determinants of spatial variation in LD risk and incidence. For example, socio-cultural factors, recreation activities, demographics and urbanism patterns influence humans and in turn, occurrence of LD cases. The times of highest entomologic risk determine when best to do vector surveillance. Though factors affecting the entomological risk are location dependent. Local weather patterns (e.g., temperature, humidity) are influential for tick survival. Potential reservoir-hosts for the North American LD system are rodents (e.g., white-footed mice, eastern chipmunks) and their abundance are strongly affected by their habitat variables including abundant food, forest cover and nesting site conditions. These factors affect the reservoir host-tick-human interaction and consequently cause a great deal of variation in the distribution of LD within endemic zones.

For future implications, efforts to model climate change with more sophisticated remote sensing data will improve our understanding of LD transmission cycles, identify risk areas and assess their characteristics. Using satellite imagery, geographic information systems, and spatial statistical methods in conjunction with ground-verifying ecologic studies and LD case surveillance data is a promising development in LD research. Since LD transmission depends on complex ecological systems involving more than one agent, vector, host and regionally variable, spatial analysis of LD should adopt complementary approaches to geography, GIS and spatial epidemiology from many other disciplines including ecology, entomology, zoology, climatology, and virology.

Future research should also focus on long-term data collection which provides wide coverage of environmental and climatic, biotic and abiotic variables and consequently contribute to the progress made in identifying the determinants of spatial variation in LD risk. The geographical and annual variation in the timing of human Lyme disease can be largely explained by weather conditions. Many GIS and RS based spatial models are developed for this. As availability of seasonal (temporal) and long term spatial data increases, the quality and accuracy of GIS and RS methods improve, so does the effectiveness of spatial analysis.

The significant variability in seasonal as well as spatial risk of exposure to LD within small, but ecologically diverse geographic areas shows that temporally dynamic and spatially explicit models are needed to assess the risk of exposure to tick-borne pathogens at spatial scales encompassing diverse climatologic or ecological conditions. Scale matters in predictive models. Variables with small spatial variance (*i.e.*, macro-climatic conditions) in small areas have nearly no predictive value, whereas diversified variables (*i.e.*, vegetation type) have limited value in large-scale studies. This aspect highlights the significance of multi-resolution analysis and long term monitoring studies for possible lagged climatic effects on the geographic distribution of vector populations. We need high resolution (finer scale such as Zip Codes) human incidence data to help reveal isolated endemic areas. New GIS and RS-based studies are needed to monitor occurrence at the macro-level, and GPS-based field studies help pinpoint areas of occurrence at the micro-level where spread within populations of reservoir hosts, clusters of infected ticks and tick-to-human transmission may be better understood.

Modeling risk based on habitat alone without follow-up data on the distribution of vectors and human cases would not be valid without testing the adequacy of these variables. Therefore, robust field studies are needed to validate and refine the risk maps. These risk maps can lead to new endemic areas. Surveillance methods could be targeted for tick vectors in these expanded regions. Spatial models that use coarse scales and general climate and vegetation indexes fail to capture the complex relationship between tick activity and its field environment [75]. Ground-verifying ecologic studies and acarological follow-up studies are crucial for effective control measures. Laboratory studies and spatial entomological and ecological risk models might show clear relationships between climatic variables (*i.e.*, relative humidity) and tick survival but follow-up field studies might produce conflicting results. Host abundance patterns might have not been accounted for within field studies and could provide a limitation for verification of findings [76].

For future applications, more efficient control measures could be implemented with the aid research outcomes from spatial analysis. GIS and spatial analysis could take a role in the optimal distribution strategies of the vaccines, such as locating bait stations containing a pesticide-delivery system. To reduce vector-tick populations and human-tick encounters, by means of host-targeted methods, new GIS-based suitability models should be developed for effective vaccine deployment. Future developments will further enhance the novel use of GIS merging data from various sources into an end-product tailored specifically to vaccine deployment.

Spatially comprehensive studies are needed on strategic implementation of the intervention in LD endemic areas. An important conclusion pertaining to human populations considers that people living in areas where LD was not thought to be endemic may also be at risk for infection. New prevalence studies should be conducted on newly identified areas of endemicity.

Policies directing public health objectives in minimizing risk from LD could also include partnering with the tourism sector in disease surveillance by monitoring and reporting field conditions at high risk recreation areas. More inter-sectoral initiatives could be established to assess health implications of other sectors’ climate change policies, such as urban planning, transport, energy supply, food production and water resources.
